# Implications for Efficacy and Safety of Total Dose and Dose-Intensity of Neoadjuvant Gemcitabine-Cisplatin in Muscle-Invasive Bladder Cancer: Three-Week Versus Four-Week Regimen

**DOI:** 10.3233/BLC-211556

**Published:** 2022-03-11

**Authors:** Karin Holmsten, Lise Høj Omland, Anne Birgitte Als, Mads Agerbæk, Line Hammer Dohn, Henriette Lindberg, Niels Viggo Jensen, Andreas Carus, Mette Moe, Abolfazl Hosseini, Cecilia Radkiewicz, Helle Pappot, Anders Ullén

**Affiliations:** a Department of Oncology-Pathology, Karolinska Institute, Stockholm, Sweden; b Department of Oncology, Capio Sankt Görans Hospital, Stockholm, Sweden; c Department of Oncology, Rigshospitalet, Copenhagen, Denmark; d Department of Oncology, Aarhus University Hospital, Aarhus, Denmark; e Department of Oncology, Herlev and Gentofte Hospital, Herlev, Denmark; f Department of Oncology, Odense University Hospital, Odense, Denmark; g Department of Oncology, Aalborg University Hospital, Aalborg, Denmark; h Department of Pelvic Cancer, Karolinska University Hospital, Stockholm, Sweden; i Department of Medical Epidemiology and Biostatistics, Karolinska Institutet, Stockholm, Sweden

**Keywords:** Neoadjuvant chemotherapy, muscle invasive bladder cancer, cisplatin, gemcitabine

## Abstract

**BACKGROUND:**

Neoadjuvant cisplatin-based chemotherapy is standard care prior to radical cystectomy in patients with muscle-invasive bladder cancer (MIBC).

**OBJECTIVE:**

To assess efficacy and safety of two commonly used neoadjuvant schedules with different total doses and dose-intensities of gemcitabine and cisplatin (GC).

**METHODS:**

Data were collected retrospectively from all patients treated between 2010 and 2018 with neoadjuvant chemotherapy according to clinical routine at seven centres in Sweden and Denmark. Patients in Sweden received three cycles of a 4-week schedule (GC-4w: cisplatin 70 mg/m^2^ day 1, gemcitabine 1000 mg/m^2^ days 1, 8, 15, q 28 days) and in Denmark four cycles of a 3-week schedule (GC-3w: cisplatin 70 mg/m^2^ day 1, gemcitabine 1000 mg/m^2^ days 1, 8, q 21 days). Primary endpoint was pathological response at cystectomy (pT0N0 and < pT2N0).

**RESULTS:**

A total of 251 patients were treated with GC-4w and 455 with GC-3w. pT0N0 was significantly higher for patients treated with GC-3w compared to GC-4w, 46% versus 32% (adjusted odds ratio [aOR] 1.80; 95% confidence interval [CI] 1.16–2.80; *P* = 0.009); and for < pT2N0 60% versus 47% (aOR 1.08; 95% CI 0.70–1.66; *P* = 0.743). There were no significant differences between GC-4w and GC-3w regarding survival parameters. GC-3w patients discontinued treatment more frequently and showed a higher degree of neutropenia.

**CONCLUSIONS:**

A significantly higher complete response-rate was observed in the patient group treated with the more cisplatin-dose-intense 3-week schedule. The side-effect profile was in favor of the 4-week approach while relapse-free and overall survival were similar.

## INTRODUCTION

Radical cystectomy is standard curative treatment for muscle-invasive bladder cancer (MIBC). Neoadjuvant cisplatin-based chemotherapy reduces the risk of death by 10–16% and increases the absolute overall survival (OS) at 5 years by 5% compared to cystectomy alone [1–4]. Methotrexate, vinblastine, adriamycin, and cisplatin (MVAC) has been studied in randomised trials and is one standard neoadjuvant regimen [5]. Gemcitabine and cisplatin (GC) is also commonly used in the neoadjuvant setting [6–9], extrapolated from results in metastatic urothelial cancer (mUC) where GC shows similar OS but a milder toxicity profile compared to MVAC [10]. GC demonstrates response rates (pT0N0 and < pT2N0) and survival benefit in the same range as with MVAC [11]. Different schedules of MVAC and GC have been used, varying in numbers of cycles, dose intensities and total doses, but the optimal neoadjuvant regimen remains undefined.

To our knowledge, no studies in the neoadjuvant setting have compared GC regimens with different dose-intensities and total doses of cisplatin and gemcitabine. In the present study we compared treatment patterns, toxicity, downstaging efficacy, and survival of a 4-week schedule (GC-4w) with a more cisplatin-dose-intense 3-week schedule (GC-3w).

## METHODS

### Study design

This trial was conducted as a multicentre retrospective cohort study at two centres in Stockholm, Sweden, and five in Denmark, all associated with the Nordic Urothelial Cancer Oncology Group (NUCOG). The trial was approved by the Ethical Review Board Stockholm, Sweden (2013/664-31/3, 2016/1089-32, 2020-00616), and the Danish Patient Safety Authority (3-3013-3078/1). Written informed consent from the patients was waived by the ethical committees, due to the retrospective study design.

### Patients

All patients receiving neoadjuvant GC according to clinical routine between January 2010 and June 2018 at the participating centres were included. The start of data collection was chosen to match with the national decisions to initiate use of neoadjuvant chemotherapy as standard of care in the two countries (2010 in Stockholm, Sweden, and 2013 in Denmark).

Sweden and Denmark apply similar guidelines for neoadjuvant chemotherapy prior to radical cystectomy, recommending treatment to patients with histologically confirmed pure or mixed transitional cell carcinoma of the bladder, stage cT2–T4aN0M0, Eastern Cooperative Oncology Group performance status (ECOG PS) 0–1, glomerular filtration rate (GFR) ≥50 ml/min in Sweden and ≥60 ml/min in Denmark, biological age ≤75 years, and no comorbidity contradicting chemotherapy or radical cystectomy.

### Treatment

Patients were treated in accordance with routine clinical practice with three cycles of GC-4w (cisplatin 70 mg/m^2^ day 1, gemcitabine 1000 mg/m^2^ days 1, 8, and 15, q 28 days) in Stockholm, Sweden, and four cycles of GC-3w (cisplatin 70 mg/m^2^ day 1, gemcitabine 1000 mg/m^2^ days 1 and 8, q 21 days) in Denmark. Granulocyte colony-stimulating factor (G-CSF) was used according to the physician’s choice. Radical cystectomy was performed at one centre in Stockholm, Sweden and at five centres in Denmark, all being high volume centres [12]. Robotic surgery was used as standard technique at five of the six centres. Extended lymphadenectomy was performed to the aortic bifurcation except at one of the Danish centres, where dissection extended only to the ureter crossing.

### Outcome

The primary endpoint was pathological response at cystectomy: pT0N0 and < pT2N0. Secondary endpoints were relapse rate, relapse-free survival, bladder-cancer-specific survival, overall survival, toxicity, and treatment patterns.

Response was defined as pathological complete response (pT0N0) and downstaging to non-muscle-invasive disease (< pT2N0 = pT0N0, pTisN0, pTaN0, or pT1N0) versus residual muscle-invasive or node-positive disease (pT2–pT4a and/or pN1-3). Partial response was defined as pT1N0, pTaN0, or pTisN0. Patients receiving curative radiotherapy or not undergoing cystectomy were excluded from the primary pathological response analyses.

Relapse-free survival was calculated as time from the start of neoadjuvant chemotherapy to the date of relapse (radiological or pathological), last follow-up, or death, whichever occurred first. Bladder-cancer-specific survival and overall survival were measured from start of neoadjuvant chemotherapy until date of bladder-cancer-specific death or death from all causes or last follow-up, respectively. Patients who died from perioperative cystectomy-related complications were considered as bladder-cancer-specific deaths. All patients were included in the survival analysis.

Treatment related toxicity, grade 3 and 4 adverse events (AEs), was assessed according to the National Cancer Institute’s Common Terminology Criteria for Adverse Events (NCI-CTCAE) version 5.0. The clinical stage was assessed according to the TNM classification (the Union for International Cancer Control [UICC] 8th edition 2016) [13]. The WHO Classification of Tumours of the Urinary System and Male Genital Organs 2016 was used for pathological grading of the cystectomy specimens [14].

### Statistical analysis

Continuous variables were presented as medians and ranges and categorised to be assessed using the Pearson χ2 test. Significance was set at *P* < 0.05. Odds ratios (ORs) were estimated with 95% confidence intervals (CIs) using logistic regression models. To estimate adjusted odds ratios (aORs), multivariate logistic regression including baseline characteristics was applied.

To contrast relapse rate, bladder-cancer-specific mortality, and overall mortality in the two treatment arms, flexible parametric models were used to estimate hazard ratios (HRs) with 95% CIs within 3 years from the start of chemotherapy [15]. Adjusted hazard ratios (aHRs) included baseline characteristics. The Kaplan-Meier method was used to illustrate the effect of treatment on survival. Further standardised survival curves were fitted by applying flexible parametric models including the baseline characteristics allowing for the effect of treatment to vary over follow-up.

As a sensitivity analysis, excess death was estimated (corresponding to bladder-cancer-specific survival) by using a relative survival framework comparing the overall mortality in GC-4w- and GC-3w-treated MIBC patients with the overall mortality in the Swedish and Danish populations, respectively. Expected survival in the two populations matched by age, sex, and year of chemotherapy start were estimated using the Ederer II method. Five-year relative survival was defined as the ratio of the observed (patient) to the expected (population) survival using a cohort approach [16]. GC-4w-to-GC-3w crude and adjusted HRs within 3 years from start of chemotherapy were estimated using flexible parametric models [15].

Data were analysed using SPSS statistics software for Windows (version 26; IBM SPSS, Armonk, NY, USA) and Stata Intercooled 15.1 (StataCorp LP, College Station, TX, USA).

## RESULTS

### Baseline characteristics

Of the 706 patients included in the study, 251 were treated with GC-4w and 455 with GC-3w (Table 1). Median follow-up time was 3.6 years in the GC-4w and 2.7 years in the GC-3w group. Significant differences in baseline characteristics at diagnosis were observed between the two treatment schedules: patients receiving GC-4w were more frequently included earlier in the study period, presented with better ECOG PS but lower GFR, and had significantly more advanced clinical T stage.

**Table 1:** Baseline characteristics

Characteristics	GC-4w *n* = 251	GC-3w *n* = 455	*P*-value
Treatment calendar period
2010–2012	80 (32)	5 (1)
2013–2015	102 (41)	219 (48)
2016–2018	69 (28)	231 (51)	< 0.005
Age, years
Median (range)	67 (44–80)	65 (34–79)
Age interval
34–59 years	46 (18)	115 (25)
60–69 years	121 (48)	205 (45)
70–80 years	84 (34)	135 (30)	0.104
Sex
Male	184 (73)	324 (71)
Female	67 (27)	131 (29)	0.553
ECOG PS
0	237 (94)	345 (76)
1	14 (6)	70 (15)
Missing data	0	40 (9)	< 0.005
GFR, ml/min		160;
Median (range)	82 (32–134)	90 (40–172)
GFR interval
< 60 ml/min	32 (13)	4 (1)
≥60 ml/min	219 (87)	451 (99)	< 0.005
Clinical T stage
cT1	0	1 (0)
cT2	110 (44)	237 (52)
cT3	117 (47)	48 (11)
cT4a	24 (10)	11 (2)
cT2–cT4a^a^	0	158 (35)	< 0.005
Clinical Nstage
cN0	249 (99)	449 (99)
cN1^b^	2 (1)	2 (0)
cNx	0	4 (1)	0.531

Data are *n* (%), except where indicated. ^a^Not assessable, muscle-invasive bladder cancer cT2-cT4a. ^b^Clinically suspicious lymph nodes at baseline radiology. ECOG PS, Eastern Cooperative Oncology Group performance status; GFR, glomerular filtration rate.

### Treatment

Treatment patterns are presented in Table 2. The mean number of cycles delivered were 2.7 for GC-4w and 3.3 for GC-3w. Eighty percent of the GC-4w patients received all three planned cycles of treatment, whereas only 60% of the GC-3w patients received the planned four cycles (*P* < 0.005). The main reasons for stopping GC-3w treatment prematurely were decreased kidney function (9%), impaired hearing (6%), and neutropenia (5%). Few patients discontinued neoadjuvant chemotherapy due to progressive disease: 5% for GC-4w and 2% for GC-3w. Detailed treatment patterns are summarised in Supplementary Table 1. Dose delays were significantly more common in the GC-3w than in the GC-4w patients (27% versus 6%, *P* < 0.005). Neutropenia was by far the most common reason for dose delays in the GC-3w group, causing 73% of the dose delays compared to only 7% in the GC-4w cohort.

**Table 2:** Treatment patterns and adverse events

	GC-4w *n* (%)	GC-3w *n* (%)	OR (95% CI)	*P*-value	aOR (95% CI)^a^	*P*-value
Treatment patterns
Patients with
Interrupted treatment	51 (20)	184 (40)	2.66 (1.86–3.82)	< 0.005	2.57 (1.58–4.20)	< 0.005
Dose delay^b^	14 (6)	125 (27)	6.41 (3.60–11.42)	< 0.005	4.89 (2.23–10.73)	< 0.005
Dose reduction^b^	92 (37)	73 (16)	0.33 (0.23–0.47)	< 0.005	0.44 (0.27–0.70)	< 0.005
Omitted dose^b^	132 (53)	105 (23)	0.27 (0.19–0.38)	< 0.005	0.29 (0.19–0.45)	< 0.005
Any treatment modification^b^	187 (75)	306 (67)	0.70 (0.50–0.99)	0.045	0.57 (0.36–0.90)	0.016
G-CSF^b^	20 (8)	122 (27)	4.23 (2.56–6.99)	< 0.005	1.73 (0.91–3.29)	0.097
Adverse events (AEs) grade 3/4^c,d^
Haematological AEs
Anaemia	30 (12)	36 (8)
Neutropenia	89 (36)	199 (44)	1.42 (1.03–1.95)	0.032	1.18 (0.77–1.82)	0.444
Febrile neutropenia	9 (4)	16 (4)
Thrombocytopenia	50 (20)	89 (20)	0.98 (0.66–1.44)	0.908	0.89 (0.54–1.48)	0.663
≥1 any grade 3/4 haematological AE	116 (46)	227 (50)	1.16 (0.85–1.58)	0.350	0.87 (0.57–1.32)	0.510
Non-haematological AEs
Infection	16 (6)	21 (5)
Thromboembolic event	15 (6)	24 (5)
Decreased renal function	7 (3)	15 (3)
Impaired hearing	1 (0)	11 (2)
Peripheral neuropathy	2 (1)	8 (2)
Heart failure	0	5 (1)
Non-specified	15 (6)	45 (10)
≥1 any grade 3/4 non- haematological AE	49 (20)	119 (26)	1.46 (1.00–2.13)	0.048	1.51 (0.90–2.52)	0.118

^a^Adjusted for calendar period, age, sex, ECOG, GFR, and clinical stage. ^b^In at least one cycle. ^c^More than one AE per patient possible. ^d^AEs with outcome less than 10% are excluded from the logistic regression model due to too few events. G-CSF, granulocyte colony stimulating factor; OR, odds ratio; aOR, adjusted odds ratio; CI, confidence interval.

The numbers of dose reductions and omitted doses were significantly higher with the GC-4w than the GC-3w regimen. However, for cisplatin on day 1, the degree of dose reduction in at least one cycle was higher for GC-3w than for GC-4w (14% versus 7%, *P* = 0.005). For gemcitabine day 15, a substantial proportion of the patients in the GC-4w group had a dose reduction (30%) or an omitted dose (47%) in at least one cycle, most often due to thrombocytopenia or neutropenia.

Median time from last infusion of neoadjuvant chemotherapy to cystectomy was 4.7 weeks for GC-4w and 5.0 weeks for GC-3w (Supplementary Table 1). Ten patients received curative radiotherapy, and eleven patients did not undergo the curative intended radical cystectomy (Table 3). Six patients died due to perioperative complications at cystectomy.

**Table 3:** Response and survival

Response	GC-4w *n* (%)	GC-3w *n* (%)	OR (95% CI)	*P*-value	aOR^a^ (95% CI)	*P*-value
Complete response, pT0N0	77 (32)	202 (46)	1.85 (1.33–2.57)	< 0.005	1.80 (1.16–2.80)	0.009
Partial response, < pT2N0	113 (47)	259 (60)	1.67 (1.21–2.29)	< 0.005	1.08 (0.70–1.66)	0.743
pT2-pT4 and/or N+	128 (53)	176 (41)	0.60 (0.44–0.82)	< 0.005	0.93 (0.60–1.44)	0.743
pT stage
pT0	83 (34)	211 (48)	1.80 (1.30–2.49)	< 0.005	1.79 (1.16–2.76)	0.008
pT1	15 (6)	18 (4)	0.65 (0.32–1.32)	0.237	0.48 (0.18–1.24)	0.129
pT2	43 (18)	63 (14)	0.79 (0.51–1.20)	0.263	0.88 (0.49–1.56)	0.654
pT3	60 (25)	84 (19)	0.73 (0.50–1.06)	0.097	0.81 (0.49–1.34)	0.409
pT4	20 (8)	20 (5)	0.54 (0.28–1.02)	0.056	2.43 (0.76–7.73)	0.133
pTis	21 (9)	37 (8)	0.98 (0.56–1.71)	0.942	0.36 (0.18–0.75)	0.006
pTa	3 (1)	7 (2)	1.30 (0.33–5.09)	0.702	0.69 (0.09–5.45)	0.728
Radiotherapy^b^	4 (2)	6 (1)
No cystectomy^c^	2 (1)	9 (2)
pN stage
pN0	196 (81)	376 (86)	1.46 (0.96–2.24)	0.079	1.32 (0.74–2.36)	0.347
pN1	22 (9)	31 (7)	0.76 (0.43–1.35)	0.355	0.77 (0.36–1.64)	0.498
pN2-3	23 (10)	28 (6)	0.65 (0.37–1.16)	0.145	0.76 (0.34–1.71)	0.504
Radiotherapy^b^	4 (2)	6 (1)
No lymphadenectomy^c^	6 (2)	14 (3)
	Deaths	3-year survival	Deaths	3-year survival	3-year HR (95% CI)	*P*-value	3-year aHR^a^ (95% CI)	*P*-value
Survival	*n* (%)	%	*n* (%)	%
All-cause death	86 (34)	72.6	126 (28)	73.2	0.95 (0.70–1.29)	0.730	1.36 (0.89–2.07)	0.155
Bladder cancer death	75 (30)	75.2	105 (23)	76.9	0.90 (0.65–1.25)	0.542	1.30 (0.82–2.04)	0.260
Excess death	73.8	75.7	105.9	76.6	0.92 (0.65–1.29)	0.620	1.37 (0.85–2.20)	0.200
Bladder cancer relapse	85 (34)	67.9	129 (28)	71.0	0.87 (0.65–1.16)	0.320	1.24 (0.84–1.84)	0.284

^a^Adjusted for calendar period, age, sex, ECOG, GFR, and clinical stage. ^b^Comorbidity (*n* = 4) and patients choice (*n* = 6). ^c^No cystectomy due to progressive disease (*n* = 7), comorbidity (*n* = 3), and patients choice (*n* = 1). No lymphadenectomy (*n* = 9). OR, odds ratio; aOR, adjusted odds ratio; CI, confidence interval; HR, hazard ratio; aHR, adjusted hazard ratio.

### Adverse events

Table 2 presents grade 3/4 AEs. The proportion of patients with grade 3/4 neutropenia was significantly larger in the GC-3w than the GC-4w group (44% versus 36%, *P* < 0.005). G-CSF was accordingly used significantly more often in GC-3w than in GC-4w treatment (27% versus 8%, *P* < 0.005). No difference in renal toxicity was observed between the two schedules.

### Efficacy

Pathological response data are summarised in Table 3. pT0N0 and < pT2N0 were significantly more frequent with GC-3w than with GC-4w: pT0N0 was achieved in 46% of GC-3w patients compared to 32% of GC-4w patients (OR 1.85; 95% CI 1.33–2.57; *P* < 0.005), and the corresponding numbers for < pT2N0 were 60% and 47% (OR 1.67; 95% CI 1.21–2.29; *P* < 0.005). The significant difference in downstaging to pT0N0 was still valid after adjusting for the imbalances in baseline characteristics between the two treatment cohorts. Furthermore, clinical stage ≤ cT3N0, and treatment in the earlier calendar periods were associated with higher rates of pT0N0 (Supplementary Table 2).

**Fig. 1:**
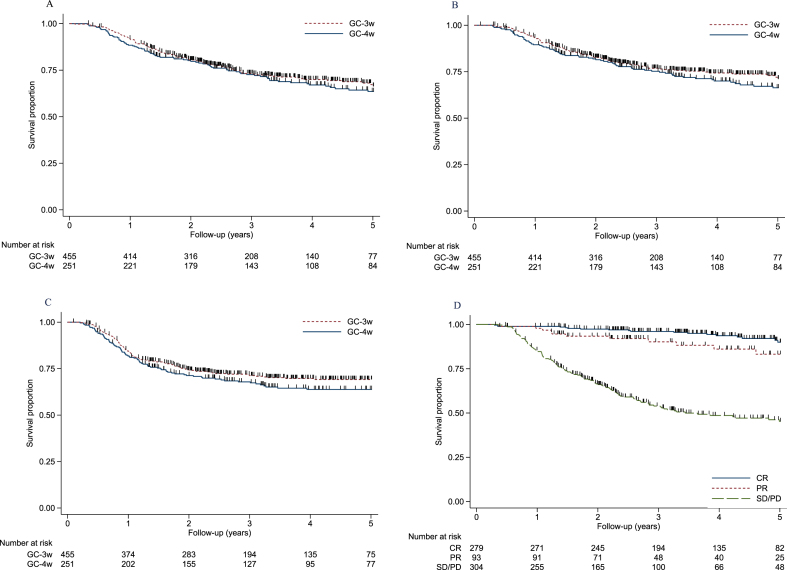
Overall survival (A), bladder-cancer-specific survival (B), and relapse-free survival (C) for GC-4w versus GC-3w. (D) Bladder-cancer-specific survivalby pathological response: CR, complete response (pT0N0); PR, partial response (pT1N0, pTisN0, pTaN0); and SD/PD, stable or progressive disease (≥pT2N0/N+).

There was no significant difference in relapse rate between GC-4w and GC-3w. In both groups, the vast majority (84%) of relapses were diagnosed within two years after the start of neoadjuvant chemotherapy (Supplementary Table 3 and Fig. 1C). The median time from relapse to death was short, 6.2 months for GC-4w and 5.1 months for GC-3w.

**Fig. 2:**
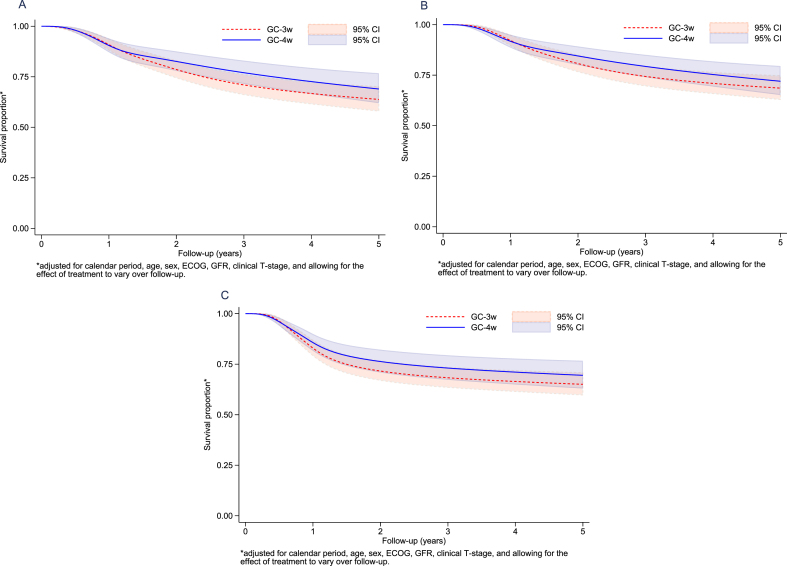
Adjusted overall survival (A), adjusted bladder-cancer-specific survival (B), and adjusted relapse-free survival (C) for GC-4w versus GC-3w standardized for calendar period, age, sex, ECOG, GFR, and clinical T-stage, allowing for the effectof treatment to vary overthe follow-up period.

The significant difference in complete response rate between GC-4w and GC-3w did not translate into different survival outcomes (Table 3 and Fig. 1A–C). Analysis of excess death by use of a relative survival framework taking into account differences in background mortality in Sweden and Denmark yielded estimates and survival curves that were very similar to those obtained when applying a bladder-cancer-specific approach. Survival proportions and HRs remained non-significant after adjusting for baseline characteristics (Table 3 and Fig. 2A–C). In the flexible parametric model, female sex, and clinical stage cT3–cT4aN0 implied a poorer survival (Supplementary Table 4). For the total cohort, the 5-year OS rate was 65%, and patients with complete response and partial response had significantly better OS rates than patients with pathological remaining muscle-invasive or node-positive disease; 5-year survival rates were 90%, 83%, and 45%, respectively (Fig. 1D). Patients with complete response in GC-4w and in GC-3w had high 5-year survival rate, 85% respectively 92%, see Supplementary Figure 2A and B. Patients with positive lymph-nodes at cystectomy (any pT-stage) had a poor 5-year overall survival rate (25%) compared to patients without lymph-node involvement (73%) (Supplementary Figure 1A and B).

## DISCUSSION

Gemcitabine in combination with cisplatin is one commonly used neoadjuvant regimen in MIBC. In this study, we compared two GC schedules, GC-4w and GC-3w, that differ regarding cumulative doses and dose intensity of both gemcitabine and cisplatin. Patients treated with GC-3w regimen showed a significantly higher degree of pathological response (pT0N0 and < pT2N0) compared to patients treated with GC-4w. The higher dose intensity and cumulative dose of cisplatin (280 versus 210 mg/m^2^) can plausibly explain the larger proportion of pT0N0 in patients receiving GC-3w. Similarly, in the recently published neoadjuvant phase III trial VESPER, ddMVAC with a higher total dose and dose intensity of cisplatin showed significantly higher pathological downstaging compared to a 3-week schedule of GC [17]. The pathological downstaging rate for the GC-3w regimen in our trial is comparable with or exceeds the best downstaging data reported in MIBC for MVAC and GC, including dose-dense regimens [5, 11, 17–20].

Pre-treatment clinical stage (cTNM) has been shown to be an important prognostic factor for pathological downstaging (pTNM) at cystectomy [11, 21]. In the present study, the GC-4w-treated patients had more advanced tumours (cT3–cT4aN0) at baseline. After adjusting for the imbalance in the pre-treatment clinical stage, the GC-3w patients still achieved pT0N0 more frequently, although residual confounding cannot be ruled out. Moreover, patients with cT3–cT4aN0 in both cohorts had significantly lower rates of complete and partial response compared to patients with pre-treatment clinical stage cT2N0.

Interestingly, the favourable downstaging efficacy in the patient group treated with GC-3w did not however translate into a corresponding improvement in relapse-free, bladder-cancer-specific, or overall survival. Sensitivity analysis using a relative survival framework that took differences in background mortality in Sweden and Denmark into account confirmed the robustness of our data, and it yielded survival estimates that were nearly identical to those obtained when using bladder-cancer-specific mortality. Patients achieving pathological complete response showed 5-year survival of 90%, confirming that pT0N0 is a prognostic marker for favourable outcome [5, 22, 23].

The present study demonstrates the importance of a cisplatin-dose-intensive chemotherapy regimen to maximise the downstaging efficacy of the primary tumour in the bladder. However, no statistical differences were detected in relapse rate or survival parameters between GC-4w and GC-3w, indicating a similar proportion of patients with disseminated micro-metastatic disease which presumably was *de novo* resistant to GC. Considering efficacy in eradicating distant micro metastases in MIBC, it is plausible that the sensitivity of individual tumour cells to cisplatin is more important than the final cumulated dose and dose intensity of cisplatin. To further improve the efficacy of GC as neoadjuvant treatment, it appears important to combine GC with drugs active on cisplatin resistant tumor cells rather than to further explore more dose-intense GC-combinations. This can be done by for example adding immunotherapy with immune checkpoint inhibitors (ICIs) [24–26], targeted therapies such as inhibitors of fibroblast growth factor receptor (FGFR) [27], or antibody-drug conjugates (ADCs) targeting Nectin-4 [28]. For patients with remaining residual muscle-invasive or node-positive disease the prognosis was poor (45% 5-year survival rate) an observation in line with previous studies [5, 22, 23]. Novel approaches for these patients, i.e., adjuvant precision-based treatment based on the biomarker profiles in the cystectomy specimen or in liquid biopsies, are warranted [29, 30].

The GC-3w schedule was associated with a higher degree of grade 3/4 AEs and patients treated with this regimen also more frequently discontinued treatment and experienced dose delays, mainly due to a significantly higher incidence of neutropenia. These findings indicate that G-CSF prophylaxis should be considered as a routine treatment as part of the GC-3w regimen. In the GC-4w arm, a low dose intensity was seen in gemcitabine day 15, which is in line with results from comparison of the two schedules in mUC [31]. Non-haematological grade 3/4 AEs (including decreased renal function, impaired hearing, and peripheral neuropathy) were few in both treatment groups, however grading of side effects are known to be underestimated in retrospective studies [32].

The main strengths of the present trial are the large size of the total cohort of consecutively treated patients and that criteria for neoadjuvant chemotherapy are similar in Stockholm, Sweden, and Denmark. Furthermore, the 3-week and 4-week GC schedules are standard of care in the two countries, thereby avoiding selection bias in the choice of chemotherapy regimens. The main limitation is the retrospective non-randomised approach, with the risk of bias from unknown cofounders and/or residual confounding despite careful adjustments. Median follow-up was relatively short and longer follow-up may allow for more accurate estimates on OS. Moreover, we lacked information regarding the extent of the diagnostic TUR-B and surgical cystectomy outcomes (i.e., number of lymph nodes resected, positive surgical margins, and type of urinary diversion).

In conclusion, the patient group treated with neoadjuvant chemotherapy with a more cisplatin-dose-intense 3-week regimen showed a significantly higher complete pathological response-rate compared to a commonly used 4-week gemcitabine-cisplatin schedule. The toxicity profile was manageable in both treatment regimens, but more neutropenia and premature treatment termination was observed in association to the GC-3w regimen. Relapse-free and overall survival were similar, indicating that future prospective studies should focus on identifying novel perioperative combination regimens which are active on cisplatin-gemcitabine resistant micro-metastatic disease.

## Supplementary Material

Supplementary Figure 1

Supplementary Figure 2

Supplementary Tables
