# Supplementary material for: Implications for Efficacy and Safety of Total Dose and Dose-Intensity of Neoadjuvant Gemcitabine-Cisplatin in Muscle-Invasive Bladder Cancer: Three-Week Versus Four-Week Regimen
Source: Bladder Cancer. 2022 Mar 11;8(1):71–80. doi: 10.3233/BLC-211556 (PMC11181847; doi:10.3233/BLC-211556)
Supplement: Supplementary Figure 2 [file blc-8-blc211556-s002.docx]

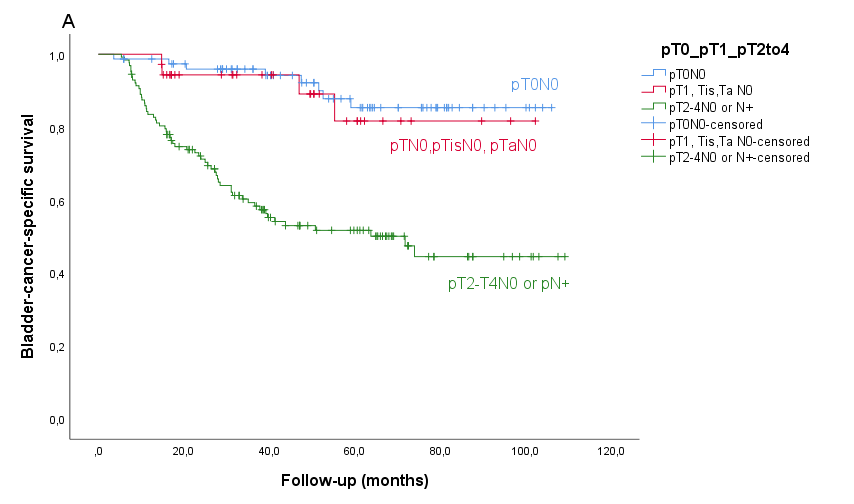

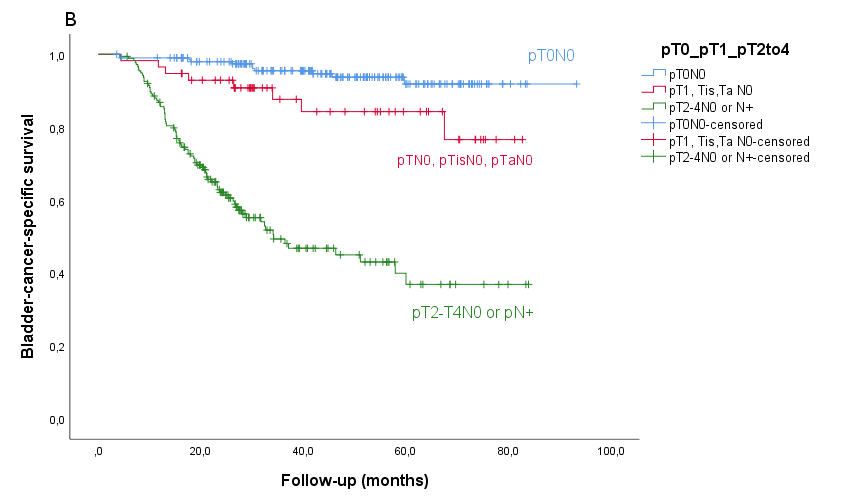


Supplementary Fig. 2. Bladder-cancer-specific survival by pathological response in GC-4w (A), and in GC-3w (B).
