# Supplementary material for: Implications for Efficacy and Safety of Total Dose and Dose-Intensity of Neoadjuvant Gemcitabine-Cisplatin in Muscle-Invasive Bladder Cancer: Three-Week Versus Four-Week Regimen
Source: Bladder Cancer. 2022 Mar 11;8(1):71–80. doi: 10.3233/BLC-211556 (PMC11181847; doi:10.3233/BLC-211556)
Supplement: Supplementary Tables [file blc-8-blc211556-s003.docx]

| Supplementary Table 1  Treatment patterns | | | |
| --- | --- | --- | --- |
| Characteristic | GC-4w  *n* = 251 | GC-3w  *n* = 455 | *P*-value |
| Median number of cycles (range)  Mean number of cycles  No. of cycles  1  2  3  4  No. of treatment-occasions with cisplatin delivered of total planned (%)  No. of treatment-occasions with gemcitabine delivered of total planned (%) | 3 (1–3)  2.7  18 (7)  33 (13)  200 (80)  -  91  80 | 4 (1–4)  3.3  48 (11)  55 (12)  81 (18)  271 (60)  82  78 |  |
| Time, median (range), weeks  start chemotherapy to cystectomy  stop chemotherapy to cystectomy | 13.9 (4.1–31.1)  4.7 (1.1–26.1) | 14.4 (3.0–36.1)  5.0 (1.9–26.1) |  |
| No. of patients who stopped treatment prematurely  Reason to stop treatment prematurely^b^  Anaemia  Neutropenia  Thrombocytopenia  Febrile neutropenia  Infection  Decreased kidney function  Thromboembolic event  Impaired hearing  Peripheral neuropathy  Heart failure  Progressive disease  Other reasons | 51 (20)  0  4 (2)  5 (2)  5 (2)  8 (3)  8 (3)  5 (2)  2 (1)  0  0  13 (5)  14 (6) | 184 (40)  4 (1)  21 (5)  6 (1)  4 (1)  6 (1)  42 (9)  10 (2)  28 (6)  14 (3)  3 (1)  7 (2)  8 (2) | < 0.005 |
| No. of patients with dose delay^a^  Reason for dose delay^b^  Anaemia  Neutropenia  Thrombocytopenia  Neutropenic fever  Infection  Decreased kidney function  Other reasons | 14 (6)  1 (0)  1 (0)  0  1 (0)  9 (4)  1 (0)  2 (1) | 125 (27)  1 (0)  91 (20)  10 (2)  4 (1)  7 (2)  6 (1)  15 (3) | < 0.005 |
| No. of patients with G-CSF^a^ | 20 (8) | 122 (27) | < 0.005 |
| No. of patients with any omitted dose^a^  No. of patients with omitted gemcitabine day 8^a^  Reason omitted day 8^b^  Anaemia  Neutropenia  Thrombocytopenia  Neutropenic fever  Infection  Decreased kidney function  Other reason  No. of patients with omitted gemcitabine day 15^a^  Reason omitted day 15^b^  Anaemia  Neutropenia  Thrombocytopenia  Neutropenic fever  Infection  Decreased kidney function  Other reason | 132 (53)  50 (20)  0  10 (4)  4 (2)  1 (0)  13 (5)  7 (3)  30 (12)  119 (47)  6 (2)  55 (22)  70 (28)  4 (2)  16 (6)  5 (2)  26 (10) | 105 (23)  105 (23)  0  34 (7)  5 (1)  1 (0)  12 (3)  13 (3)  47 (10)  - | < 0.005  0.332 |
| No. of patients with any dose reduction^a^  No. of patients with cisplatin dose reduction day 1^a^  Reason for cisplatin dose reduction day1^b^  Anaemia  Neutropenia  Thrombocytopenia  Neutropenic fever  Infection  Decreased kidney function  Other reason  No. of patients with gemcitabine dose reduction day 8^a^  Reason for gemcitabine dose reduction day 8^b^  Anaemia  Neutropenia  Thrombocytopenia  Neutropenic fever  Infection  Decreased kidney function  Other reason  No. of patients with gemcitabine dose reduction day 15^a^  Reason for gemcitabine dose reduction day 15^b^  Anaemia  Neutropenia  Thrombocytopenia  Neutropenic fever  Infection  Decreased kidney function  Other reason | 92 (37)  17 (7)  0  5 (2)  5 (2)  0  0  6 (2)  6 (2)  34 (14)  0  16 (6)  18 (7)  0  0  5 (2)  6 (2)  75 (30)  1 (0)  19 (8)  64 (25)  0  0  4 (2)  4 (2) | 73 (16)  63 (14)  1 (0)  17 (4)  7 (2)  4 (1)  0  5 (1)  32 (7)  56 (12)  1 (0)  27 (6)  8 (2)  5 (1)  0  1 (0)  19 (4)  - | < 0.005  0.005  0.637 |
| Data are *n* (%), except where indicated.  ^a^In at least one cycle.  ^b^More than one reason per patient possible. | | | |

| Supplementary Table 2  Multivariable analysis of factors predicting pathologic response | | | | | | | | |
| --- | --- | --- | --- | --- | --- | --- | --- | --- |
|  |  | pT0N0 | |  |  | < pT2N0 | |  |
|  | OR (95% CI) | | *P*-value | | OR (95% CI) | | *P*-value | |
| Chemotherapy regimen  GC-4w  GC-3w | 1  1.80 (1.16–2.80) | | 0.009 | | 1  1.08 (0.70–1.66) | | 0.743 | |
| Treatment calendar period  2010–2012 | 1 | |  | | 1 | |  | |
| 2013–2015 | 0.83 (0.46–1.47) | | 0.517 | | 1.31 (0.74–2.29) | | 0.355 | |
| 2016–2018 | 0.53 (0.29–0.97) | | 0.038 | | 0.92 (0.51–1.66) | | 0.781 | |
| Age interval  34–59 years | 1 | |  | | 1 | |  | |
| 60–69 years | 1.01 (0.68–1.51) | | 0.959 | | 1.00 (0.67–1.49) | | 0.994 | |
| 70–80 years | 1.29 (0.83–2.00) | | 0.261 | | 1.30 (0.84–2.03) | | 0.244 | |
| Sex  Male  Female | 1  0.86 (0.60–1.22) | | 0.398 | | 1  0.67 (0.48–0.96) | | 0.027 | |
| ECOG performance status  0 | 1 | |  | | 1 | |  | |
| 1 | 0.68 (0.41–1.24) | | 0.145 | | 0.57 (0.35–0.94) | | 0.028 | |
| Missing data | 0.90 (0.45–1.79) | | 0.755 | | 1.33 (0.63–2.80) | | 0.454 | |
| GFR  < 60 ml/min | 1 | |  | | 1 | |  | |
| ≥ 60 ml/min | 2.23 (0.86–5.76) | | 0.098 | | 2.38 (1.02–5.57) | | 0.045 | |
| cTNM  cT2N0 | 1 | |  | | 1 | |  | |
| cT3N0 | 0.65 (0.42–1.01) | | 0.054 | | 0.44 (0.29–0.67) | | < 0.005 | |
| cT4N0 | 0.33 (0.13–0.83) | | 0.019 | | 0.26 (0.11–0.60) | | < 0.005 | |
| cTxN0 | 0.91 (0.61–1.37) | | 0.657 | | 1.10 (0.72–1.68) | | 0.661 | |
| ECOG, Eastern Cooperative Oncology Group; GFR, glomerular filtration rate; OR, odds ratio; CI, confidence interval. | | | | | | | | |

| Supplementary Table 3  Relapse | | | | | | |
| --- | --- | --- | --- | --- | --- | --- |
|  | GC-4w | GC-3w |  |  |  |  |
|  | *n* (%) | *n* (%) | OR (95% CI) | *P*-value | aOR^a^ (95% CI) | *P***-**value |
| Relapse | 85 (34) | 129 (28) | 0.77 (0.56–1.08) | 0.127 | 1.38 (0.86–2.21) | 0.177 |
| Relapse during year 1 | 45 (53) | 68 (54) |  |  |  |  |
| Relapse during year 2 | 25 (29) | 41 (33) |  |  |  |  |
| Relapse during year 3 | 7 (8) | 9 (7) |  |  |  |  |
| Relapse after year 3 | 8 (9) | 7 (6) |  |  |  |  |
|  |  |  |  |  |  |  |
| Time from chemo start  to relapse, months |  |  |  |  |  |  |
| Median (range) | 11.4 (2.5–65.2) | 11.3 (2.3–66.9) |  |  |  |  |
| Time from relapse to  death, months |  |  |  |  |  |  |
| Median (range) | 6.2 (0.03–39.0) | 5.1 (0.2–28.0) |  |  |  |  |
| Data are *n* (%), except where indicated.  ^a^Adjusted for calendar period, age, sex, ECOG, GFR, and clinical stage.  OR, odds ratio; aOR, adjusted odds ratio; CI, confidence interval. | | | | | |  |

| Supplementary Table 4  Multivariable analysis of factors predicting survival | | | |
| --- | --- | --- | --- |
|  | All-cause survival | Bladder-cancer-specific survival | Relapse-free survival |
|  | HR (95% CI) | HR (95% CI) | HR (95% CI) |
| Chemotherapy regimen |  |  |  |
| GC-4w | 1 | 1 | 1 |
| GC-3w | 1.36 (0.89–2.07) | 1.30 (0.82–2.04) | 1.24 (0.84–1.84) |
| Treatment calendar period |  |  |  |
| 2010–2012 | 1 | 1 | 1 |
| 2013–2015 | 0.87 (0.53–1.43) | 0.86 (0.51–1.46) | 0.70 (0.44–1.09) |
| 2016–2018 | 0.79 (0.47–1.35) | 0.81 (0.46–1.43) | 0.72 (0.45–1.16) |
| Age interval |  |  |  |
| 34–59 years | 1 | 1 | 1 |
| 60–69 years | 1.00 (0.68–1.45) | 0.93 (0.63–1.38) | 0.82 (0.58–1.15) |
| 70–80 years | 0.90 (0.59–1.37) | 0.79 (0.50–1.24) | 0.73 (0.50–1.08) |
| Sex |  |  |  |
| Male | 1 | 1 | 1 |
| Female | 1.66 (1.22–2.26) | 1.77 (1.27–2.46) | 1.81 (1.36–2.42) |
| ECOG performance status |  |  |  |
| 0 | 1 | 1 | 1 |
| 1 | 1.24 (0.79–1.93) | 1.22 (0.76–1.96) | 1.30 (0.86–1.95) |
| missing | 1.12 (0.59–2.10) | 0.82 (0.38–1.79) | 0.83 (0.42–1.66) |
| GFR |  |  |  |
| < 60 ml/min | 1 | 1 | 1 |
| ≥ 60 ml/min | 0.58 (0.32–1.04) | 0.60 (0.32–1.13) | 0.54 (0.31–0.94) |
| cTNM |  |  |  |
| cT2N0 | 1 | 1 | 1 |
| cT3N0 | 1.48 (1.01–2.18) | 1.62 (1.07–2.45) | 1.37 (0.95–1.96) |
| cT4N0 | 2.13 (1.18–3.85) | 2.41 (1.29–4.49) | 1.97 (1.13–3.44) |
| cTxN0 | 0.87 (0.57–1.33) | 1.04 (0.66–1.62) | 0.90 (0.60–1.34) |
| HR, hazard ratio; CI, confidence interval; ECOG PS, Eastern Cooperative Oncology Group performance status; GFR, glomerular filtration rate. | | | |
